# Pediatric autoimmune hepatitis shows a disproportionate decline of regulatory T cells in the liver and of IL-2 in the blood of patients undergoing therapy

**DOI:** 10.1371/journal.pone.0181107

**Published:** 2017-07-11

**Authors:** Jana Diestelhorst, Norman Junge, Jerome Schlue, Christine S. Falk, Michael P. Manns, Ulrich Baumann, Elmar Jaeckel, Richard Taubert

**Affiliations:** 1 Department of Gastroenterology, Hepatology and Endocrinology, Hannover Medical School, Hannover, Germany; 2 Pediatric Gastroenterology and Hepatology, Department of Paediatric Kidney, Liver and Metabolic Diseases, Hannover Medical School, Hannover, Germany; 3 Department of Pathology, Hannover Medical School, Hannover, Germany; 4 Institute of Transplantation Immunology and Integrated Research and Treatment Center Transplantation (IFB-Tx), Hannover Medical School, Hannover, Germany; Universite Paris-Sud, FRANCE

## Abstract

**Background & Aims:**

The autoimmune hepatitis (AIH) is a chronic hepatitis driven by the adaptive immunity that affects all age groups. A functional and numerical regulatory T cell (Treg) defect has been reported in pediatric AIH (pAIH), while an intrahepatic increase in adult AIH (aAIH) patients has been detected in current research findings.

**Methods:**

Therefore, we quantified the intrahepatic numbers of Treg, T and B cells, as well as serum cytokine levels before and during therapy in pAIH.

**Results:**

We found a disproportional intrahepatic enrichment of Tregs in untreated pAIH compared to pediatric non-alcoholic fatty liver disease. The increase of Treg/total T cells was even more pronounced than in aAIH due to fewer infiltrating T and B cells. Portal densities of Treg, as well as total T and B cells, declined significantly during therapy. However, portal Treg densities decreased disproportionately, leading to even decreasing ratios of Treg to T and B cells during therapy. Out of 28 serum cytokines IL-2 showed the strongest (10fold) decrease under therapy. This decline of IL-2 was associated with decreasing intrahepatic Treg numbers under therapy. None of the baseline T and B cell infiltration parameters were associated with the subsequent treatment response in pAIH.

**Conclusions:**

Intrahepatic Tregs are rather enriched in untreated pAIH. The disproportional decrease of Tregs during therapy may be caused by a decrease of IL-2 levels. New therapies should, therefore, aim in strengthening intrahepatic immune regulation.

## Introduction

Autoimmune hepatitis (AIH) is a chronic immune mediated hepatitis that manifests in all age groups and is currently displaying an increase of incidences [1]. In contrast to adult AIH (aAIH), the pediatric form (pAIH) often manifests more acutely and has a more aggressive disease course [2]. In pAIH patients the proportions of AIH type 2 (AIH-2), that is characterized by the presence of antibodies against Cytochrome P450 2D6 and/or Formimidoyltransferase Cyclodeaminase, and biliary autoimmune manifestation, the so called autoimmune sclerosing cholangitis are higher [3]. Furthermore, the frequencies of the major genetic risk alleles of the MHC class II locus are age dependent. While both HLA-DRB1*03 and *04 predispose to adult manifestations, pAIH is associated with HLA-DRB1*03 and HLA-DRB1*13, relative to the geographical region [2, 4, 5].

T cells are supposed to be main drivers of the autoimmune response in AIH by the innate and adaptive immune system [6, 7]. With the first opportunity to detect human regulatory T cells on the basis of CD4^+^CD25^+/high^, a reversible numerical and functional defect of Treg was suspected in the peripheral blood; mostly in pAIH [8–10]. With the discovery of more specific human Treg markers like CD127, FOXP3 and its methylation status these results could not be confirmed in the blood and livers of adult AIH patients. Intrahepatic studies rather demonstrated an accumulation of Treg in untreated AIH compared to various control cohorts of inflamed and non-inflamed livers [11–14]. Moreover, liver infiltration of Treg in aAIH declined under therapy disproportionately, compared to total T and B cell numbers especially in those adult patients who did not reach biochemical remission [13].

During the reassessment of peripheral blood Treg in pediatric autoimmune liver diseases (AIH and autoimmune sclerosing cholangitis) with the latest Treg markers a numerical Treg defect but no stringent functional defect in suppression assays could be confirmed [15]. However, the T cell compartment in the peripheral blood is not necessarily representative of the intrahepatic milieu. Neither the intrahepatic Treg accumulation in aAIH nor any Treg infiltration pattern during various scenarios after adult liver transplantation was reasonably reflected in the peripheral blood [11, 13, 16–19].

Until the start of this study there was no systematic analysis of intrahepatic Treg numbers available, neither before nor under ongoing therapy in pAIH. Thus, we retrospectively immunophenotyped the intrahepatic T and B cell compartment, including Treg in pAIH in the initial diagnosis and in the follow-up during therapy.

## Material and methods

### Patients

We retrospectively included all pediatric patients with biopsy proven AIH of our clinic between 2001 and 2015. Children with evidence for autoimmune sclerosing cholangitis, replicative viral hepatitis, an AIH score below 10 [20], and any ongoing treatment with immunosuppressive medication during the initial diagnosis of pAIH were excluded from the study (Table 1, S1 Fig) [4]. Additionally, 13 available pAIH follow-up biopsies under therapy between 1996 and 2012 were included. Pediatric non-alcoholic fatty liver disease (pNAFLD, Table 1) was used as a pediatric non-autoimmune liver disease comparator. Healthy pediatric liver tissue was not available for ethical reasons. Adult AIH patients, at the time of diagnosis and during therapy, were derived from our previous studies [13].

**Table 1:** Patient data.

	Pediatric AIH		Pediatric NAFLD (for liver histology)	Pediatric control (for blood cytokines)
	At diagnosis	Under therapy		
Number of biopsies	40	13	12	34
Patient age at biopsy [yrs]	13.1 (5.4)	16.7 (6.9)	14.4 (5.1)	11.9 (6.0)
Gender [female/male]	30/10	9/4	5/7	23/11
AIH score at diagnosis	19.0 (4.0)			
mHAI	7.0 (4.9)	5.0 (5.5)	1.0 (1.9)	
Time since diagnosis [yrs]		4.0 (4.9)		
**Laboratory tests**				
Alanine aminotransferase [times ULN]	9.3 (12.1)	4.6 (10.9)	1.9 (1.2)	0.41 (0.2)
Aspartate aminotransferase [times ULN]	10.0 (17.3)	2.6 (5.1)	1.3 (0.8)	0.86 (0.3)
Gamma-glutamyl transferase [times ULN]	3.3 (6.2)	1.2 (2.6)	0.7 (2.6)	0.25 (0.1)
Alkaline phosphatase [times ULN]	1.2 (1.0)	0.5 (0.5)	0.6 (0.3)	0.65 (0.3)
Bilirubin [times ULN]	1.0 (2.6)	0.8 (1.0)	0.6 (0.9)	0.38 (0.2)
Prothrombin time [%]	71.0 (23.0)	76.0 (31.3)	93.0 (15.5)	88.0 (15.8)
Immunoglobulin G [times ULN]	1.7 (1.1)	1.0 (1.5)	0.8 (0.3)	0.72 (0.3)
**Immunosuppression prior to biopsy**				
Prednisolone		8/13 (61.5%)		
Budesonide		2/13 (15.4%)		
Azathioprine (AZA)		13/13 (100%)		
Steroid + AZA		10/13 (76.9%)		
Steroid + AZA + Tacrolimus		1/13 (7.7%)		
**Treatment response**				
Biochemical remission		3		
- Histologic remission		2		
Incomplete response		9		

The pediatric control group for cytokines analyses was age and gender matched to pAIH at diagnosis as far as possible (Table 1). These children mostly exhibited abdominal discomfort, constipation or diarrhea, without the evidence of a liver, autoimmune, coeliac, or inflammatory bowel disease. The utilization of their blood samples was approved by the local research Ethics Committee of the Hannover Medical School.

Complete biochemical remission (BR) was defined as a persistent normalization of aminotransferases (ALT, AST) and immunoglobulin G (IgG) upon standard therapy with steroids (prednisolone or budesonide) and/or azathioprine [21]. Incomplete biochemical response (IR) was defined as an improvement of ALT, AST and IgG without normalization under standard therapy over at least 2 years duration.

This study was approved by the local research Ethics Committee of the Hannover Medical School. Written informed consent was obtained from all parents and/or guardians of each child with pAIH and pNAFLD participating in this study.

### Histology

Formalin fixed and paraffin embedded liver biopsies were processed as described [13, 17]. Histological examination and scoring for the modified hepatitis activity index (mHAI) [22] were performed by an experienced liver pathologist in a blinded fashion. Intrahepatic immunophenotyping was performed in a blinded fashion and FOXP3 staining was confirmed by a second blinded observer.

**Fig 1:**
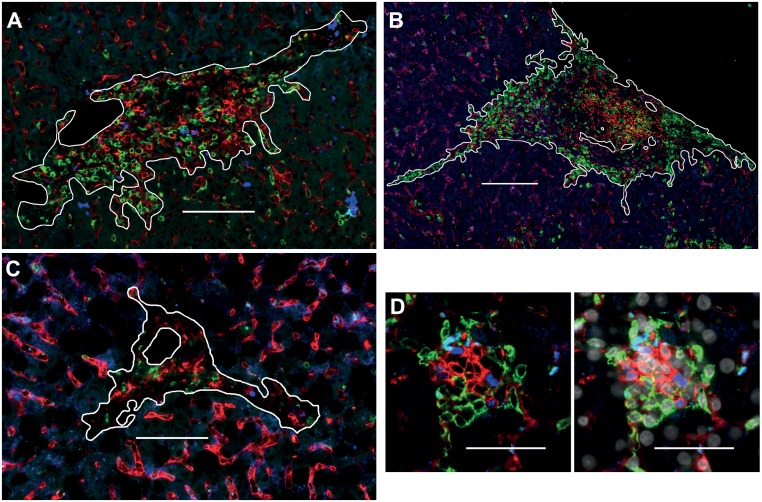
Multicolor immunofluorescence of formalin-fixed and paraffin embedded liver biopsies from pAIH. **(A)** T cell staining with CD4 (red), CD8 (green), FOXP3 (blue) in a single formalin fixed and paraffin embedded liver biopsy section with pediatric AIH and **(B)** B cell staining with CD79a (red) and CD4 (green, autofluorescence in blue) in subsequent liver biopsy sections. The white lines surround the evaluated area of the portal infiltrates and exclude lumen of veins, arteries and bile ducts. White bars represent 100 μm. **(C)** T cell staining of comparator liver biopsies with pediatric non-alcoholic fatty liver disease as in A. **(D)** Surface expression of CD4 (red) and CD8 (green) in comparison to nuclear colocalization of FOXP3 (blue) and DAPI (white) in pediatric AIH. White bars represent 50 μm.

We analyzed the intrahepatic infiltration density of CD4^+^CD8^-^FXOP3^-^, CD8^+^CD4^-^FOXP3^-^, CD4^+^CD8^-^FOXP3^+^, CD8^+^CD4^-^FOXP3^+^ and CD79a^+^ (pan B cell marker) in portal derived liver infiltrates (Fig 1A–1D). Lobular infiltrations cannot be normalized to the infiltration area as is the case with portal infiltrates and is biased by normal lymphocytic passage through sinusoids.

### Detection of cytokines in human sera

Cytokine concentrations in patients sera were quantified by multiplex protein arrays, according to the manufacturer’s instruction (BioRad Laboratories, USA) as described [13]. In brief, a 2-laser array reader (Bio-Plex, BioRad Laboratories) simultaneously quantifies all cytokines of interest. Standard curves and concentrations were calculated with Bio-Plex Manager 4.1.1 on the basis of the 5-parameter logistic plot regression formula. Bio-Plex Pro Human Cytokine 27-Plex and Bio-Plex Pro Human Cytokine 27-Plex (BioRad Laboratories) were used to detect IL-1b, IL-1RA, IL-2, IL-4, IL-5, IL-6, IL-7, IL-8, IL-9, IL-10, IL-12(p70), IL-13, IL-15, IL-17, Eotaxin, FGFb, G-CSF, GM-CSF, IFNg, IP-10, MCP-1, MIP-1a, PDGF, MIP-1b, RANTES, TNFa, VEGF, HGF.

### Statistical analysis

Statistical analysis was performed with SPSS 23.0 and GraphPad Prism 5. The Mann-Whitney U test was used for comparison of two and the Kruskal-Wallis test with Dunn post hoc test for comparisons of more than two groups. Correlation analyses were calculated with the Spearman’s rank correlation coefficient. P-values below 0.05 (two-tailed) were considered statistically significant in all analyses.

## Results

### Detection of intrahepatic Tregs in pediatric liver biopsies

We detected CD4, CD8, and FOXP3 simultaneously in a multicolor immunofluorescence in formalin-fixed paraffin embedded liver biopsies. This technique reliably detects Tregs as CD4^+^CD8^-^FOXP3^+^ cells (in the following CD4^+^FOXP3^+^; Fig 1). This histological Treg quantification could be validated by flow cytometry (CD4^+^CD25^+^CD127^low^FOXP3^+^) and by the methylation status of Treg specifc demethylation region (TSDR) of the FOXP3 gene [13, 17, 23, 24]. This is important, because activated effector T cells express FOXP3 too [23, 25]. Unfortunately, pediatric liver biopsies were too miniscule for the isolation of sufficient amounts of genomic DNA analysis of the TSDR. In the absence of an epigenetic validation for this pediatric cohort the frequency of CD8^+^CD4^-^FOXP3^+^ serves as a control for the contamination of activated T cells in the pool of FOXP3^+^ cells. However, this frequency is rather low in this pediatric cohort (CD8^+^CD4^-^FOXP3^+^/(CD8^+^CD4^-^FOXP3^+^+CD4^+^CD8-FOXP3^+^ = 3.7%) and similar low frequencies of CD8^+^CD4^-^FOXP3^+^ were found in the adult cohorts, in which we could perform the epigenetic validation with the TSDR analysis (2.8% for AIH and 3.8% for acute cellular rejection) [13, 17].

### Intrahepatic Tregs are enriched in untreated pAIH

**Fig 2:**
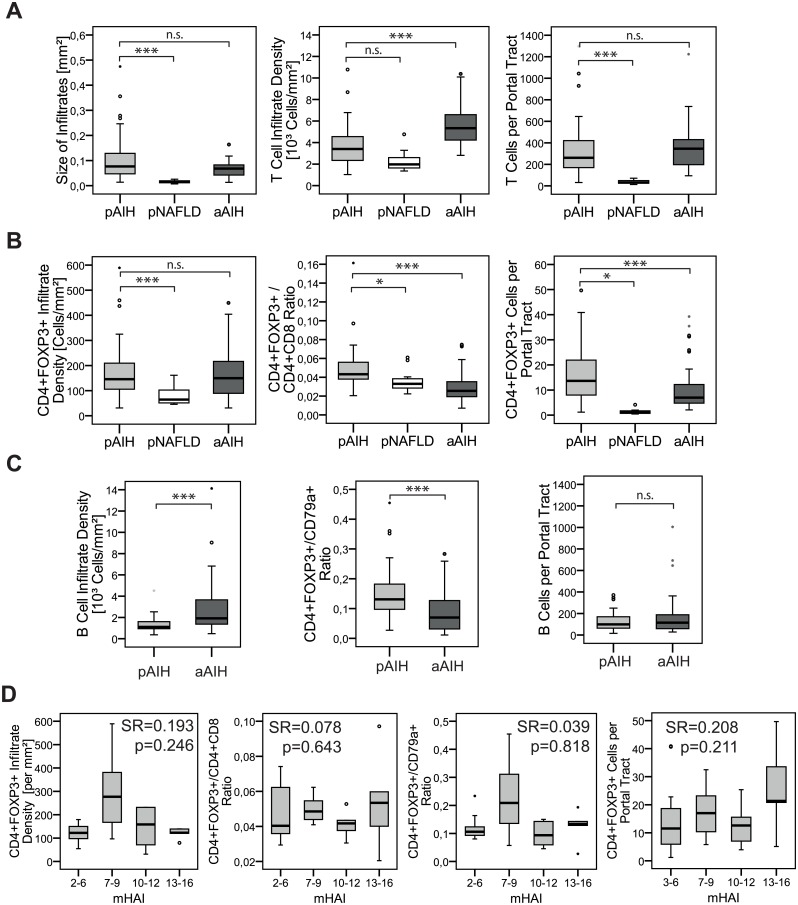
Portal T cell infiltration pattern in untreated AIH-1. **(A-C)** Comparison of size of portal infiltrates, portal cell densities and portal cell ratios, as well as absolute numbers per portal tract of T cells (CD4^+^+CD8^+^), B cells (CD79a^+^) and CD4^+^FOXP3^+^ Treg in untreated pediatric AIH (pAIH; n = 40), pediatric non-alcoholic liver disease (pNAFLD; n = 12) and untreated adult AIH (aAIH; n = 45). **(D)** Correlation analysis (n = 38; SR: Spearman rank correlation coefficient) of the portal CD4^+^FOXP3^+^ Treg cell densities and cell ratios as well as the absolute number per portal tract with the hepatitis activity index (mHAI). Horizontal line represents the median and error bars the interquartile range. (* p<0.05; ** p<0.01; *** p<0.001; n.s.: not significant)

To prove the hypothesis of a numerical Treg deficiency the selection of a comparator cohort is crucial. In the absence of liver samples from healthy children we chose pNAFLD as pediatric non-autoimmune liver disease control. Thereby, we selected pNAFLD liver biopsies with a portal inflammation score (Ishak D) of 0–1 to come as close to noninflamed portal tracts as possible (Fig 1C). This approach is underlined by significantly larger portal tracts in untreated pAIH compared to pNAFLD (Fig 2A). Compared to the non-autoimmune pNAFLD pAIH had significantly more portal CD4^+^FOXP3^+^ Treg, in terms of cell density and ratio to CD4^+^ as well as CD8^+^ T cells within the portal tracts and in terms of absolute numbers per portal tract (Fig 2A and 2B).

Untreated pAIH patients were found to have less dense infiltrations of CD4^+^ as well as CD8^+^ T cells and CD79a^+^ B cells compared to untreated aAIH patients (Fig 2A–2C) [13]. Although, CD4^+^FOXP3^+^ Treg infiltration densities were comparable in untreated pediatric and adult AIH the ratios of CD4^+^FOXP3^+^ Treg to T cells and B cells within the portal tracts were even higher in pAIH as compared to aAIH (Fig 2A–2C).

The numbers of patients with pAIH-2 (n = 3) were too low for a statistical comparison of the intrahepatic T and B cell infiltration between the two AIH types.

None of the portal infiltration parameters of CD4^+^, CD8^+^, CD4^+^FOXP3^+^ T cells, CD79a^+^ B cells and the infiltrate size correlated with the histological disease severity assessed by the mHAI in untreated pAIH (Fig 2D, S2 Fig).

Similar as in adults [13] serum immunoglobulin G levels were significantly correlated with the portal B cell/T cell ratio (Spearman rho (SR) = 0.366; p = 0.022),

### Therapy for pAIH caused a disproportionate decline of portal Treg infiltration

Thirteen follow-up biopsies under ongoing therapy were available for the intrahepatic immunophenotyping as in untreated pAIH (Table 1). Since relapse rates are higher in children [2] immunosuppression withdrawal is uncommon in pAIH. Hence, the majority of follow-up biopsies (10/13) wereperformed due to incomplete biochemical remission.

**Fig 3:**
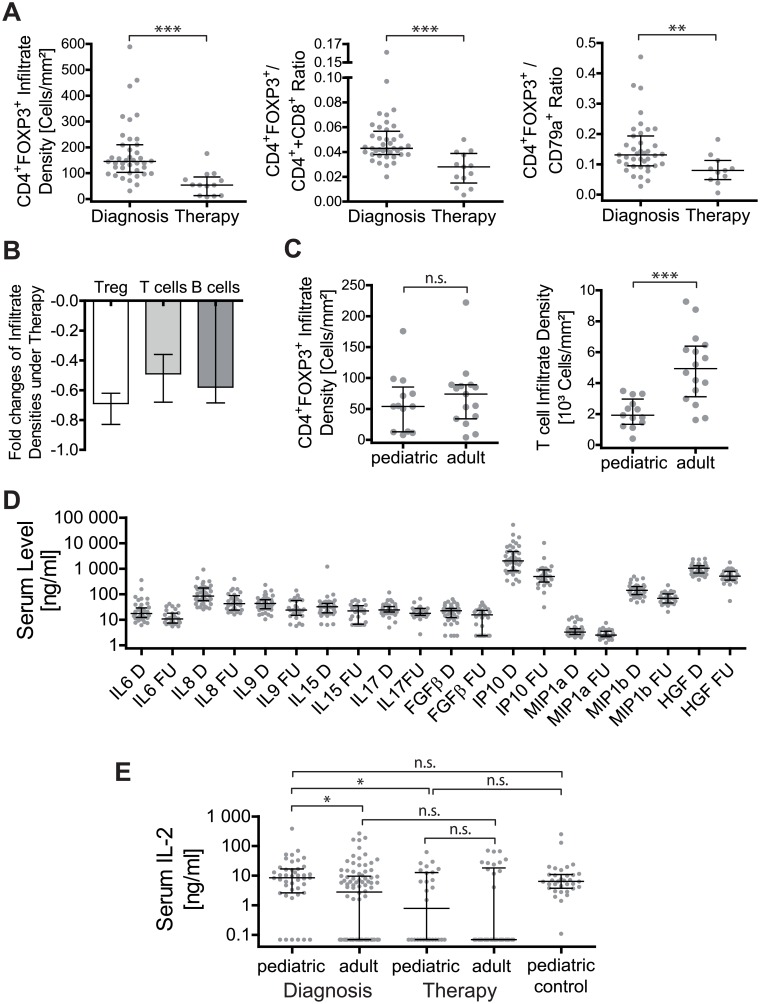
IL-2 associated decline of portal Treg infiltration under therapy for pAIH. **(A)** Comparison of portal cell densities and portal cell ratios of CD4^+^FOXP3^+^ Treg in untreated pediatric AIH (n = 40) and under ongoing therapy (n = 13). **(B)** Fold changes of CD4^+^FOXP3^+^ (Treg; n = 11), CD4^+^+CD8^+^ (T cells; n = 11) and CD79a^+^ cells (B cells; n = 9) under therapy in paired samples. **(C)** Comparison of CD4^+^FOXP3^+^ Treg and total T cells (CD4^+^+CD8^+^) in treated pediatric AIH (n = 13) and treated adult AIH matched for the treatment response (n = 16). **(D)** Quantification of serum cytokine levels in untreated pAIH at diagnosis (D; n = 43) and during follow-up (FU; n = 28) under therapy. Depicted are all cytokine with significant changes in the follow-up in paired and unpaired non-parametric comparisons. The horizontal line and error bars represent the median and the interquartile range. The seventeen cytokines without significant changes are not shown. Cytokine levels below the detection threshold were set to the lowest detected concentration. **(E)** Serum IL-2 levels in pediatric AIH similar to (D) and adult AIH (diagnosis: n = 88; under therapy: n = 34), as well as age and gender, matched pediatric controls for cytokines (n = 34). (n.s.: not significant; * p<0.05; ** p<0.01; *** p<0.001)

Similar to adults, the infiltration density of T and B cells, but not the size of infiltrates, were significantly reduced in treated compared to untreated pAIH (Table 2) [13]. Thereby, CD4^+^FOXP3^+^ Treg exhibited the strongest decline of all analyzed cell types within the portal tracts resulting in a significantly reduced portal ratio of Treg to T and B cells in therapy (Fig 3A and 3B). Due to the limited numbers of biopsies during biochemical remission (BR; 3/13) we could not compare the infiltration pattern between IR and BR as in adults [13]. The finding of similar portal Treg infiltration densities with fewer T and B cell densities in therapy in pAIH compared to aAIH persisted during therapy, when aAIH comparators were matched to include similar treatment responses as the pAIH group (adult: 13 IR + 3 randomly chosen BR from our previous study [13]; pediatric: 10 IR + 3 BR) (Fig 3C).

**Table 2:** Intrahepatic immunophenotyping of portal derived infiltrates in pediatric AIH.

	At diagnosis (median and IQR)	Under therapy (median and IQR)	p value
Number of biopsies	40	13	
mHAI	7.0 (5.5–10.5)	4.5 (1.25–7.0)	0.020
Infiltrate size [mm^2^]	0.079 (0.050–0.132)	0.045 (0.022–0.227)	0.154
CD4^+^ + CD8^+^ density [cells per mm^2^]	3373 (2338–4717)	2065 (1139–3144)	0.003
CD79^+^ density [cells per mm^2^]	1135 (946–1626)	710 (190–1070)	0.030
CD4^+^FOXP3^+^ density [cells per mm^2^]	146 (106–221)	63 (12–91)	<0.001
CD4^+^FOXP3^+^ / CD79^+^ ratio	0.129 (0.094–0.198)	0.077 (0.052–0.086)	0.004
CD4^+^FOXP3^+^ / CD4^+^+ CD8^+^ ratio	0.044 (0.040–0.059)	0.021 (0.013–0.041)	<0.001

In parallel serum levels of 28 cytokines were quantified in untreated pAIH (n = 43) and those in ongoing therapy (n = 28). None of the cytokine levels correlated with the portal lymphocyte infiltration pattern in paired blood and liver samples in untreated pAIH (data not shown). When untreated and treated blood samples were compared, the levels of 11 cytokines decreased significantly in non-parametric paired and unpaired tests in therapy (Fig 3D and 3E). Thereby IL-2, which is essential for Treg homeostasis, exhibited the strongest decline (about 10fold), while the other cytokines declined about 2–5 fold. When pAIH and aAIH (aAIH untreated: n = 88; aAIH treated: n = 34) were compared, pAIH had higher IL-2 serum level at diagnosis and aAIH only showed a trend to a decline of IL-2 during therapy (Fig 3E). Compared to none AIH controls without known liver or autoimmune diseases there was no evidence for overall reduced peripheral IL-2 levels in pAIH at diagnosis or under therapy (Fig 3E). Many samples had IL-2 levels below the detection threshold and thus their levels were set to the lowest measured concentration for subsequent non-parametric comparisons.

### Intrahepatic immunophenotyping did not predict the subsequent treatment response in pAIH

We had regular follow up visits from 32/40 patients of whom untreated biopsies were immunophenotyped. Treatment response was determined according to the AASLD guidelines [21]. Thereby patients were only classified as IR after treatment durations of at least 24 months. Shorter treatment durations without reaching BR were not classified and excluded from this analysis. Based on this approach we had 17 baseline biopsies with subsequent BR, 14 with subsequent IR, one with subsequent liver transplantation (Ltx) and 8 biopsies with insufficient treatment duration. There were no significant differences in the portal baseline infiltration of all analyzed cell types or cell ratios when we compared BR with IR or BR with IR+Ltx (Table 3).

**Table 3:** Association of intrahepatic baseline immunophenotyping and subsequent treatment response.

	Subsequent biochemical remission (median IQR)	n	Subsequent incomplete response (median IQR)	n	p value
Number of biopsies	17		15		
mHAI	6.5 (5.1–13.0)	16	7.0 (5.0–9.0)	15	0.572
Age at diagnosis [yrs]	12.86 (10.81–14.91)	17	13.42 (8.21–14.99)	15	0.970
Infiltrate size [mm^2^]	0.090 (0.037–0.207)	17	0.070 (0.045–0.099)	15	0.370
CD4^+^ + CD8^+^ density [cells per mm^2^]	3069 (2289–4410)	17	3435 (2619–4391)	15	0.628
CD79^+^ density [cells per mm^2^]	1058 (946–1609)	17	1391 (1020–2050)	14	0.316
CD4^+^FOXP3^+^ density [cells per mm^2^]	139 (110–171)	17	150 (111–232)	15	0.478
CD4^+^FOXP3^+^ / CD79^+^ ratio	0.129 (0.097–0.163)	17	0.114 (0.088–0.220)	14	0.953
CD4^+^FOXP3^+^ / CD4^+^+ CD8^+^ ratio	0.045 (0.038–0.065)	17	0.044 (0.038–0.058)	15	0.970

## Discussion

The present study closes a gap in the current controversy about a Treg defect as a driver of AIH. Previously, we and others could not confirm such a Treg deficiency in the blood and livers of aAIH patients with the application of the latest markers for human Treg (mostly FOXP3, CD127 and demethylation of TSDR of the FOXP3 gene locus) but rather consistently found an intrahepatic enrichment of Treg in untreated aAIH [11, 13, 14]. However, at least lower Treg numbers in the peripheral blood of pediatric autoimmune liver diseases could be confirmed with the latest Treg markers [15].

In contrast, Treg accumulated in the liver of untreated pAIH as it has been reported for aAIH patients [13]. Moreover, there were higher absolute numbers of Treg per portal tract and, due to lower infiltration with T- and B cells, there were even higher Treg/T cell and Treg/B cell ratios in pAIH as compared to aAIH. A parallel study from Egypt confirmed our results and described higher FOXP3^+^ cell numbers and higher FOXP3^+^/CD4^+^ ratios in the livers of pAIH compared to non-AIH liver diseases [15]. However, Behairy and colleagues analyzed single color stainings and thus cannot exclude the detection of FOXP3^+^ non-Treg like CD8^+^FOXP3^+^ cells. This may explain their higher FOXP3^+^/CD4^+^ ratio (median 0.14) compared to our CD4^+^FOXP3^+^/CD4^+^ (median: 0.079). Furthermore Behairy and colleagues only analyzed untreated but no treated pAIH biopsies.

Assuming a Treg deficiency as a driver of AIH, more severe AIH should be associated with less Treg levels or lower Treg/Teff ratios. Our and other results argue against this hypothesis in aAIH [13] and pAIH [15]. We found similar portal Treg densities in pAIH and aAIH before and during therapy. The higher cell ratios of Treg to T- and B cells in boths points of time result from a higher density of the total lymphocytes in aAIH.

The discrepancy between lower peripheral Tregs reported by others [15] and increased intrahepatic Tregs reported here could be due to a homing of Tregs into the inflamed liver. Unfortunately, there were no PBMCs available in the present retrospective study to address this question. However, similar patterns (decrease in the blood and increase in the liver) were reported e.g. in acute cellular rejection and subclinical rejection after liver transplantation [17–19]. Both are steroid sensitive liver inflammation mediated by the adaptive immune response. Such a hepatic homing is mediated by CXCR3 and CCR4 on Treg and its ligands CXCL9-11 [13, 14, 26, 27]. Additionally intrahepatic Treg numbers in aAIH but not in pAIH are associated with levels of CD31 [13].

The increase of intrahepatic Treg numbers can result from local induction and stabilization of Treg as well. The intrahepatic microenvironment is more tolerogenic most likely to prevent immune reactions against bacterial commensals and foreign food antigens from the gut. Local factors that favor Tregs in contrast to effector T cells are centrilobular hypoxia, presence of retinoic acid e.g. enrichment of stellate cells, TGFβ release from a hepatocytes and the presence of 1,25-OH vitamin D3 and short-chain fatty acids from gut microbiota [28, 29].

The major limitation of histological studies is the lack of functional data especially when suitable antibodies against functional relevant molecules are not available or very limited as in formalin-fixed and paraffin embedded biopsies. However, intrahepatic Treg from autoimmune and inflamed livers had mostly a non-exhausted memory phenotype, expressed more functional relevant surface markers than peripheral blood Treg, and were functional in in-vitro studies [13, 14, 26]. The demethylated TSDR of the FOXP3 gene characterizes stable and suppressive Treg [23]. The histological Treg detection applied here is stringently correlated with a demethylated TSDR and thus suggests that the intrahepatic Treg in AIH have at least in principal stable functional capacities [13, 17]. Biopsy specimens were too small to perform a TSDR methylation status in pAIH. In addition, other groups could not stringently confirm a functional defect of peripheral blood Treg in pediatric autoimmune liver diseases [15].

IL-2 is essential for Treg homeostasis and survival [30, 31]. A most recent in-vitro analysis of liver explants suggested a disproportionate low IL-2 secretion in inflamed liver tissue. Furthermore, the Treg from these inflamed livers were stable but more prone to Fas-mediated apoptosis, which could be rescued by IL-2 [26]. The results of similar peripheral IL-2 levels in untreated pAIH as well as in age and gender matched no AIH controls without evidence for liver and autoimmune diseases do not imply an IL-2 deficiency in pAIH. A possible explanation may be that studies of liver explants with advanced cirrhosis are probably biased towards refractory disease under adequate immunosuppressive therapy. We found lower intrahepatic Treg counts in aAIH patients with IR compared to those with BR under ongoing therapy [13]. Due to the higher efforts for liver biopsies in children like anesthesia, we had insufficient biopsy numbers during BR to confirm this finding in pAIH. The cytokine measurement in the serum mirrors the intrahepatic milieu by an overspill into the periphery only in a limited way. However, the strong reduction of the serum IL-2 and ten other cytokines, observed here, go simultaneously to a contraction of the intrahepatic T cell pool, the main intrahepatic IL-2 source, as expressed by declining T cell densities within the shrinking infiltrates under therapy in pAIH and aAIH. This may contribute to the disproportionate decline of intrahepatic Treg. The synopsis of Chen et al. [26] and our results suggest the hypothesis of an insufficient intrahepatic Treg mediated immune regulation related to IL-2 deprivation in the chronic phase of AIH patients in whom BR is not achieved during immunosuppressive therapy. The disproportional decline of intrahepatic Treg could also be caused by the mostly steroid and azathioprine based immunosuppressive therapy. While the effect of steroids on Tregs remains unclear in vivo, and antimetabolites seem to be compatible with Treg homeostasis [32]. In contrast calcineurin inhibitors, the main second line therapy for refractory AIH, disadvantage Treg e.g. by reducing the IL-2 secretion of conventional T cells. To circumvent this imbalance of the immune regulation but retain the immunosuppressive effect on conventional T cells, combinations of calcineurin and mTOR inhibitors already entered the clinic mostly after transplantation. Additionally, combinations with low dose IL-2 could restore Treg homeostasis in experimental settings [32].

Additionally, chemokines like CXCL10 (IP10) that are involved in Treg homing into the liver are significantly downregulated under ongoing therapy in pAIH. Thus a reduced hepatic recruitment of Treg could contribute to the Treg decline under therapy as well.

Although the intrahepatic T cell numbers are associated with the remission status, the baseline T cell infiltration is not predictive for the subsequent treatment response in pAIH and aAIH [13].

In summary, there is no evidence for a numerical intrahepatic Treg defect over all age groups in human AIH. The disproportional decline of intrahepatic Treg during ongoing therapy is consistent in pAIH and aAIH and may result from a decrease of the Treg survival factor IL-2. Such associations are relevant for the design and evaluation of novel approaches for second line therapies or the withdrawal of the immunosuppressive therapies.

## Supporting information

S1 Fig Age and mHAI grades at diagnosis of untreated pediatric AIH. (A) The age distribution of the patients at the diagnosis of pAIH. (B) Scoring of the mHAI of the liver biopsies prior to treatment.
(EPS)

S2 Fig Portal T and B cell infiltration pattern in untreated pAIH. Correlation analysis (SR = Spearman rank correlation coefficient) of the size of portal infiltrates, portal cell densities and absolute numbers per portal tract of T (CD4^+^+CD8^+^) and B cells (CD79a^+^) with hepatitis activity index (mHAI) in untreated pAIH.
(EPS)
